# Synergistic Effects of Traffic-Related Air Pollution and Exposure to Violence on Urban Asthma Etiology

**DOI:** 10.1289/ehp.9863

**Published:** 2007-03-22

**Authors:** Jane E. Clougherty, Jonathan I. Levy, Laura D. Kubzansky, P. Barry Ryan, Shakira Franco Suglia, Marina Jacobson Canner, Rosalind J. Wright

**Affiliations:** 1 Department of Environmental Health and; 2 Department of Society, Human Development, and Health, Harvard School of Public Health, Boston, Massachusetts, USA; 3 Department of Environmental Health, Rollins School of Public Health, Emory University, Atlanta, Georgia, USA; 4 Department of Epidemiology, Harvard School of Public Health, Boston, Massachusetts, USA; 5 Channing Laboratory, Department of Medicine, Brigham and Women’s Hospital and Harvard Medical School, Boston, Massachusetts, USA

**Keywords:** childhood asthma, exposure to violence (ETV), geographic information systems (GIS), intraurban variability, nitrogen dioxide (NO_2_), social–environmental synergy, stress

## Abstract

**Background:**

Disproportionate life stress and consequent physiologic alteration (i.e., immune dysregulation) has been proposed as a major pathway linking socioeconomic position, environmental exposures, and health disparities. Asthma, for example, disproportionately affects lower-income urban communities, where air pollution and social stressors may be elevated.

**Objectives:**

We aimed to examine the role of exposure to violence (ETV), as a chronic stressor, in altering susceptibility to traffic-related air pollution in asthma etiology.

**Methods:**

We developed geographic information systems (GIS)–based models to retrospectively estimate residential exposures to traffic-related pollution for 413 children in a community-based pregnancy cohort, recruited in East Boston, Massachusetts, between 1987 and 1993, using monthly nitrogen dioxide measurements for 13 sites over 18 years. We merged pollution estimates with questionnaire data on lifetime ETV and examined the effects of both on childhood asthma etiology.

**Results:**

Correcting for potential confounders, we found an elevated risk of asthma with a 1-SD (4.3 ppb) increase in NO_2_ exposure solely among children with above-median ETV [odds ratio (OR) = 1.63; 95% confidence interval (CI), 1.14–2.33)]. Among children always living in the same community, with lesser exposure measurement error, this association was magnified (OR = 2.40; 95% CI, 1.48–3.88). Of multiple exposure periods, year-of-diagnosis NO_2_ was most predictive of asthma outcomes.

**Conclusions:**

We found an association between traffic-related air pollution and asthma solely among urban children exposed to violence. Future studies should consider socially patterned susceptibility, common spatial distributions of social and physical environmental factors, and potential synergies among these. Prospective assessment of physical and social exposures may help determine causal pathways and critical exposure periods.

The gradient of socioeconomic position (SEP) on health may be explained partly by a combination of increased contaminant exposures and greater susceptibility to their effects. Air pollution, for instance, may be higher near major roadways, power plants, and industrial sites, where property values are lower and lower-income populations reside (Graves 1988). Increased life stress among lower-SEP populations has also been proposed as a primary pathway through which SEP affects health (Gee and Payne-Sturges 2004; Morello-Frosch and Shenassa 2006).

Because of this potential spatial covariance across exposures, and because stress and pollution may influence common physiologic pathways (i.e., oxidative stress) and health outcomes (i.e., respiratory disease), stronger methods are needed to disentangle their effects and investigate synergies (Gee and Payne-Sturges 2004; O’Neill et al. 2003; Weiss and Bellinger 2006). The environmental justice literature has documented significant disproportionate contaminant exposures in minority and lower-SEP communities (Brulle and Pellow 2006), and the resultant influence on asthma exacerbation patterns (Maantay 2007). However, fewer studies have considered disproportionate susceptibility among lower-SEP populations.

Exposure to violence (ETV) has been conceptualized as a chronic urban stressor, potentially elevated in communities where pollution is higher. Chronic stress effects of episodic violence are grounded in trauma theory (e.g., post-traumatic stress), detailed elsewhere (Wright 2006). Episodic violence, post-traumatic stress (Augustyn et al. 2002; Overstreet and Braun 2000), and hyper-vigilance (Gordon and Riger 1991)—more prevalent in lower-SEP urban communities (Sampson et al. 1997)—may negatively influence health though physiologic alterations, including immune dysregulation, and behavioral pathways. Many urban caregivers, for example, restrict children’s behavior, keeping them indoors due to fear of violence (Levy et al. 2004; Wright et al. 2004b), making children more sedentary, increasing indoor exposures, and decreasing spatial autonomy that is important to development (Katz 1991).

Chronic stress has been linked to asthma exacerbations in cross-sectional (Oh et al. 2004) and prospective (Sandberg et al. 2004) population studies. Other evidence suggests a role for stress in the onset of asthma (Wright et al. 2002, 2004a). Chronic stress may influence hypothalamic–pituitary–adrenal (HPA) axis and cortisol dysregulation (Hellhammer et al. 1997; Ockenfels et al. 1995), glucocorticoid resistance (Miller et al. 2002), sympathetic–adrenal–medullary (SAM) activation, catecholamine production (Glaser and Kiecolt-Glaser 2005), immune mediator function, inflammation (Umetsu et al. 2002), and cytokine production (Chen et al. 2003; Wright et al. 2004a). Stress and pollution affect some common physiologic systems, facilitating synergistic effects; for example, psychological stress (Epel et al. 2004) and ozone (Fugisawa 2005) both affect oxidative stress pathways.

Few studies have examined the influence of stress on pollution susceptibility, though some findings suggest differential susceptibility by SEP, possibly mediated by life stress (Morello-Frosch and Shenassa 2006). Time-series studies indicate effect modification of short-term pollution exposures by SEP (Jerrett et al. 2004; Lin et al. 2004; Martins et al. 2004), though others found no significant modification (Zanobetti and Schwartz 2000). Fewer studies have considered long-term exposures, though some indicate greater associations between long-term air pollution and mortality among less-educated adults (Hoek et al. 2002; Krewski et al. 2000).

In urban settings, traffic-related air pollution may be elevated along with ETV, and previous studies have linked traffic-related air pollution to asthma exacerbation and respiratory outcomes. In the United States and Europe, children living or attending school near truck routes and highways show increased asthma and allergy symptoms (Brauer et al. 2002), hospitalizations (Edwards et al. 1994; Lin et al. 2002), allergic rhinitis (Duhme et al. 1996), and reduced lung function (Brunekreef et al. 1997). Traffic-related pollutants have also been associated with asthma development (Gordian et al. 2006; Zmirou et al. 2004).

Incorporating traffic-related air pollution into large-scale epidemiologic studies requires models linking traffic and ambient concentrations. Relationships between traffic and health have been examined using several different traffic indicators, with no consensus on which indictors best capture variability in traffic-related pollution or health outcomes in different settings. Prior studies have successfully extrapolated traffic exposures from sampling homes to larger cohorts using predictive land-use regression (LUR) models (Brauer et al. 2002; Brunekreef et al. 1997). LUR shows strong predictive power for intraurban nitrogen dioxide variability (Hochadel et al. 2006; Sahsuvaroglu and Jerrett 2004), using traffic and land use characteristics (i.e., population density, major sources).

In this study, we explore the hypothesis that a chronic stressor (here, lifetime ETV) predicts stronger associations between traffic-related air pollution exposure and childhood asthma development. We employ data from the Maternal-Infant Smoking Study of East Boston (MISSEB), a community-based prospective pregnancy cohort examining asthma, respiratory, and cognitive development. Questionnaires were administered detailing violence exposures (both witnessing and victimization) and avoidance behaviors (staying in at night, avoiding certain areas, keeping children indoors). Because the MIS-SEB did not examine air pollution, we used geographic information systems (GIS) and LUR at the neighborhood scale to retrospectively estimate pollution exposures, using monthly NO_2_ data collected over 18 years in the surrounding neighborhoods.

## Materials and Methods

**Figure 1:**
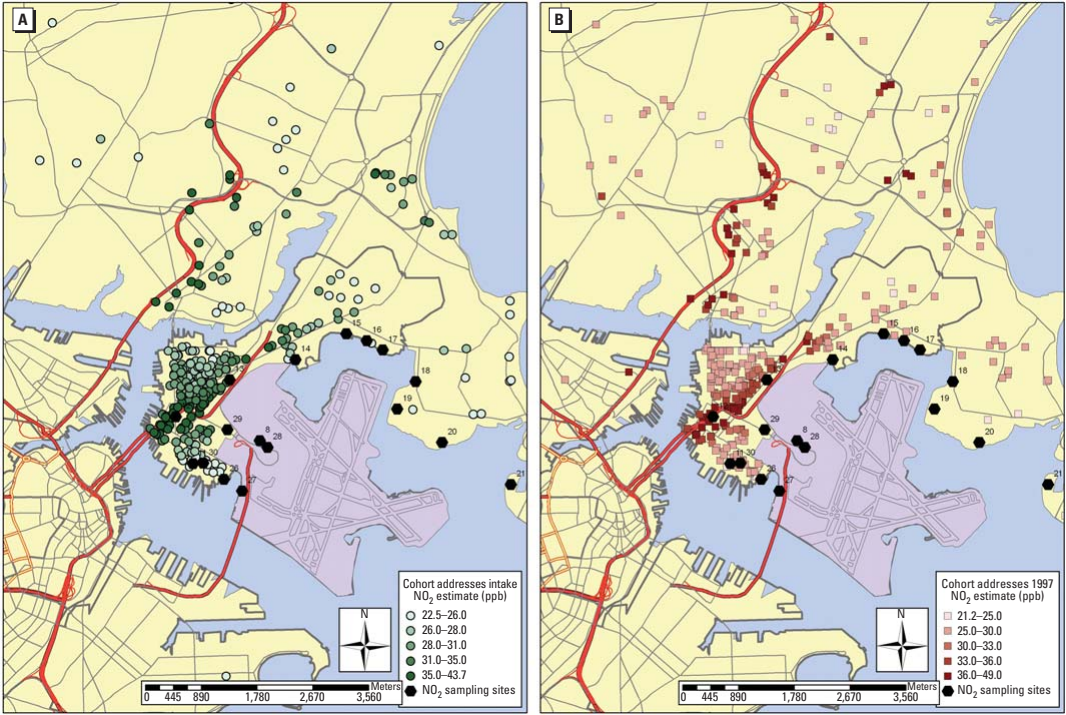
Distribution of East Boston cohort and NO_2_ sampling sites. (*A*) Residential addresses at enrollment (about 1990). (*B*) Residential addresses at violence questionnaire date (1997).

Pregnant women were recruited from East Boston Neighborhood Health Center (EBNHC), Boston, Massachusetts, between 1987 and 1993, as described elsewhere (Hanrahan et al. 1992). East Boston is a working-class urban neighborhood bisected by major highways and access roads to Logan International Airport (Figure 1). Following 888 live births originally enrolled, caregivers of 417 children completed questionnaires in 1997 detailing the child’s lifetime violence exposure. Loss to follow-up was attributed largely to families moving out of the neighborhood; those who moved but continued to participate are included. Written informed consent was obtained from participants (mothers) before study initiation, in accordance with both Brigham and Women’s Hospital and Beth Israel Deaconess Medical Center Human Subjects Committees.

### Measures

#### Traffic-related pollution exposures

NO_2_ has been shown to be a reliable indicator for traffic-related primary air pollution (Hochadel et al. 2006; Nieuwenhuijsen 2000). We used a long-term spatially resolved NO_2_ data set, explored temporal trends in pollution concentrations, compared multiple traffic indicators as predictors of concentrations, and developed retrospective exposure indices.

Passive NO_2_ samples were collected contemporaneously 1 week each month from January 1987 through December 2004, using Palmes tubes and analyzed by spectrophotometry. Samples were collected at 28 unobstructed locations, 1 m above ground, across the Logan Airport grounds and surrounding communities (Ayres 2006). We used monthly averages for 13 sites within community spaces, geocoded by hand using aerial photography. Geocoding (identifying residential locations on an active map embedded in a GIS) allowed for the analysis of spatial characteristics of each location. Missing concentrations were imputed using weighted average concentrations for surrounding months at the same site.

To explain variability in NO_2_, we considered 25 traffic indicators derived from Massachusetts Highway Department (MHD) 1990 data (Table 1) and site characteristics (land use, elevation, proximity to industrial areas, population density) derived from U.S. Census 2000 data and aerial photography (U.S. Census Bureau 2001).


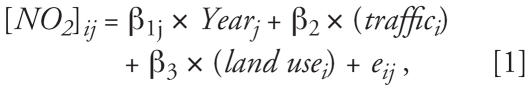


where *Year_j_* is a categorical indicator for each sampling year *j* and capturing secular pollution trends; *traffic_i_* is a suite of traffic characteristics for site *i* (candidate variables listed in Table 1); and *land use_i_* is a suite of candidate site characteristics (listed above).

Candidate variables were selected with *p* < 0.05 in univariate nonparametric associations with NO_2_. Backward elimination produced a parsimonious model with *p* < 0.1 for all terms. Because the assumption of normal distribution in NO_2_ could not be rejected (Shapiro-Wilk *p* < 0.0001), concentrations were not transformed. Models were run in PROC GLM, corroborated in PROC MIXED (SAS Institute Inc., Cary, NC) with random effects by site, to account for within-site autocorrelation.

#### Residential retrospective exposure estimates

Using the predictive NO_2_ model, we created residential exposure indices. Each child’s address at enrollment (during pregnancy) and follow-up were geocoded using the ESRI StreetMap Address Locator (ESRI, Redlands, CA), and spatial variables derived in GIS, to apply Equation 1 to each address. NO_2_ exposure estimates were defined for each year of follow-up, for both reported residences. NO_2_ exposures for years following 1997 were derived using the questionnaire address; between 1990 and 1997, lacking residential information, we created time-weighted annual exposures using the two known addresses. For example, estimated exposure in 1991 for a child enrolled in 1990 is: [(6/7) × (estimated NO_2_ in 1991 at residence reported at enrollment) + (1/7) × (estimated NO_2_ in 1991 for residence reported in 1997)].

We created this lifetime NO_2_ exposure trajectory for each child, and calculated exposures during seven intervals potentially influencing asthma etiology: *a*) life course through diagnosis (or end of follow-up); *b*) year of birth; *c*) before 5 years of age (median age of diagnosis); *d*) year of ETV questionnaire (known residential address); *e*) years between first violent event and diagnosis; *f* ) year of diagnosis; and *g*) 1 year before diagnosis.

For intervals ending at diagnosis, median diagnosis age was used for nonasthmatics. Some exposures after diagnosis (i.e., at ETV questionnaire) are considered to be attributed to the measure’s relative stability. We used univariate logistic regression for each NO_2_ interval against asthma diagnosis to select an optimal exposure interval, which is applied going forward. Sensitivity analyses on the final epidemiologic models assessed whether interval selection affected results.

#### Violence exposure assessment

Exposure to violence was assessed using the My Child’s ETV scale (Selner-O’Hagan et al. 1998). At interview, children ranged from 4 to 11.5 years of age; those > 8 years of age also answered the questionnaire themselves. The questionnaire includes items on witnessing events: *a*) hitting, slapping, punching, *b*) a shooting, *c*) a stabbing, or *d*) hearing gunshots. We added one question on witnessing domestic verbal abuse. Respondents indicate the event frequency, over their lifetime and previous year, on a scale of 1 (0–1 time) to 4 (more than 10 times). Caregivers reported child’s approximate age at first witnessing.

Rasch modeling summarized responses into a continuous score, modeling the conditional probabilities for each “yes” response, given the presumed event severity and each child’s true-but-unobserved exposure (a latent construct) (Kindlon et al. 1996). Expanding on this approach, the model was generalized to account for features theoretically influencing severity—frequency, whether events occurred at home, whether the child knew the victim(s) or perpetrator(s)—and included parent and child’s report, wherever available, as detailed elsewhere (Franco Suglia 2006).

As a validity check, we assessed the relationship between the Rasch ETV and Checklist of Children’s Distress Symptoms (CCDS) (Richters and Martinez 1990), administered contemporaneously. CCDS is a parental report of 24 post-traumatic stress symptoms over the prior 6 months (e.g., irritability, inability to fall asleep, nightmares, fear of attending school). The lifetime Rasch ETV and 6-month CCDS were significantly correlated (Spearman *r* = 0.21, *p* < 0.0001), corroborating an association between violence and distress symptoms reported elsewhere (Martinez and Richters 1993).

#### Child’s asthma diagnosis

During MISSEB follow-up, parental reports of child’s asthma diagnosis were ascertained through bimonthly telephone or face-to-face interviews over the child’s first 2 years, every 6 months through 4 years of age, and annually thereafter. Parents were asked “Since we last spoke to you, have you been told by a doctor or nurse that your child has asthma?” and likewise for asthmatic bronchitis. The same questions were asked on ETV questionnaire administration. Children were considered to have asthma if the parent reported any diagnosis of asthma or asthmatic bronchitis.

#### Potential confounders

We also ascertained demographic characteristics, smoking, and medical history through standardized questionnaires administered during MISSEB follow-up visits. Maternal education was categorized as less than high school, high school or technical school graduate, or some college. At each clinic visit during pregnancy, mothers were asked about smoking status. A urine specimen was obtained for determination of a creatinine-corrected cotinine level as detail, and mothers were classified as never-smokers during pregnancy if they always reported never smoking and their urine cotinine levels were consistently < 200 ng cotinine/mg creatinine (Hanrahan et al. 1992). Maternal asthma status was based on report of physician-diagnosed asthma using the standardized American Thoracic Society respiratory questionnaire (Ferris 1978).

### Data analysis

We examined independent associations of air pollution and ETV on asthma diagnosis using univariate odds ratios (ORs). To examine the modifying effect of violence on the NO_2_–asthma relationship, we constructed two interaction models, of the forms used elsewhere (Tsaih et al. 2004):


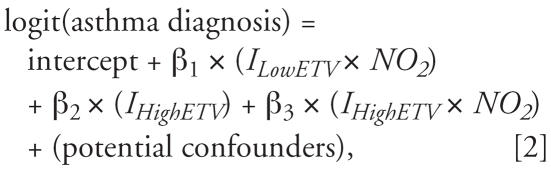


where *I_LowETV_* = 1 if below-median ETV (reference group), 0 otherwise. *I_HighETV_* = 1 if above-median ETV, 0 otherwise. *NO_2_* is a centered continuous variable with SD = 1.0; ORs thus refer to a 1-SD increase above the mean. Potential confounders included maternal asthma, education, smoking before and after pregnancy, child’s sex and age. Equation 2 produces the slopes by ETV group and their significance.

A second regression model contained main effects for ETV and NO_2_, and their interaction:


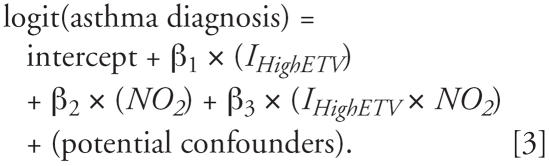


Equation 3 produces the statistical test of the interaction; if β_3_ differs significantly from zero, ETV significantly modifies the association of NO_2_ on asthma.

Because NO_2_ data were collected only in East Boston and adjoining Winthrop, we expect NO_2_ estimates to be more accurate for children always living in these neighborhoods. Likewise, the Rasch ETV likely better reflects chronic stress among children always living in the same community where exposure occurred. Therefore, all statistical analyses are performed for the entire cohort, and repeated using only lifetime residents of East Boston and Winthrop.

## Results

We were able to geocode home addresses for 409 children at follow-up, and 369 at enrollment. Demographics of those children and their caregivers are presented in Table 2. Caregivers of 100 children (24%) reported asthma diagnoses during follow-up; caregivers of an additional seven children (2%) retrospectively reported asthmatic bronchitis. Only 8% of mothers reported ever having asthma. ETV varied significantly; at least one violent event was reported for 46% of children, at least two for 18%, and Rasch scores ranged from −1.00 to 2.93. Lifetime residents did not significantly differ from the full cohort in ETV, asthma rates, or other demographic factors or confounders.

### Traffic-related pollution and LUR models

**Figure 2:**
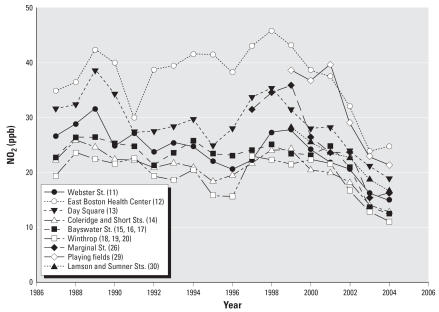
Annual average NO_2_ (ppb) across 13 neighborhood sampling sites. For legibility, average annual values at three clustered sites on Bayswater Street and in adjacent Winthrop have been combined. Numbers in the key correspond to sampling sites in Figure 1.

Over 18 years, 2,291 monthly NO_2_ samples were collected and analyzed, averaging 24.0 ppb (0.78–69.4 ppb). Some overall NO_2_ decline occurred over time, with some reordering of sites (Figure 2). LUR models explained significant variability as a function of secular trends, traffic density within 500 m, distance from major roads, and block group population density (*R*^2^ = 0.83; Table 3). More variability in NO_2_ was explained by spatial (*R*^2^ = 0.53) than temporal terms (*R*^2^ = 0.29). Residential estimates averaged 27.5 ppb (18.7–42.6 ppb) overall, higher than measured concentrations, because several NO_2_ samplers were located in open spaces (i.e., parks, backyards), whereas cohort homes clustered near major roads (Figure 1).

### Effects of NO_2_ and ETV on asthma

Univariate ORs for the seven candidate NO_2_ exposure periods indicated that a 1-SD increase only in year-of-diagnosis NO_2_ (4.3 ppb) showed near-significant associations with asthma for the full cohort [OR = 1.17; 95% confidence interval (CI), 0.94–1.46], and was used going forward as our key exposure metric.

For the full cohort, we found no independent effect of ETV on asthma (OR = 0.98; 95% CI, 0.78–1.22). Stratified analyses, however, indicated that year-of-diagnosis NO_2_ was significantly associated with asthma solely among children with above-median ETV (OR = 1.65; 95% CI, 1.16–2.34). For children with below-median ETV, there was no association between NO_2_ and asthma (OR = 0.94; 95% CI, 0.70–1.26).

Among lifetime resident children, with less expected exposure measurement error, we found similar effects with greater magnitude. There was a near-significant effect of NO_2_ on asthma (OR = 1.28; 95% CI, 0.97–1.69), with no independent effect of ETV (OR = 1.12; 95% CI, 0.84–1.48). Stratified analyses indicated that NO_2_ was significantly associated with asthma solely among children with above-median ETV (OR = 2.33; 95% CI, 1.47–3.71, vs. OR = 0.87; 95% CI, 0.59–1.28 with below-median ETV).

We used multivariate logistic regression to formally test the observed interaction, adjusting for potential confounders. In stratified analysis across the full cohort, we found elevated odds of asthma with increased NO_2_ solely among children with above-median ETV (Table 4), and the magnitude was unaffected by addition of potential confounders (adjusted OR = 1.63; 95% CI, 1.14–2.33). The interaction between NO_2_ and ETV was statistically significant (*p* = 0.03), and robust to inclusion of both main effects.

Among the lifetime residents, in multivariate logistic regression, we found similar associations with greater magnitude. Stratified analyses indicated elevated odds of asthma with increased NO_2_ solely among children with above-median ETV (adjusted OR = 2.40; 95% CI, 1.48–3.88). The NO_2_–ETV interaction term was statistically significant (*p* = 0.0009), and robust to inclusion of main effects.

Sensitivity analyses indicated that only average NO_2_ for the years between first violence exposure and asthma diagnosis could be substituted for year-of-diagnosis NO_2_ with significance (adjusted OR = 1.52; 95% CI, 1.05–2.19) for children with above-median ETV, in the full cohort multivariate model. We repeated analyses removing 10 children whose diagnosis occurred before first violent event, with little bearing on results. Last, sensitivity analyses indicated that median-dichotomized My ETV, My Child’s ETV, and CCDS scores could not be substituted for the Rasch indicator with significance.

## Discussion

We found an association between traffic-related pollution (NO_2_) and asthma diagnosis only among children with elevated ETV, after controlling for potential confounders. Of multiple exposure intervals, year-of-diagnosis NO_2_ best predicted asthma (with some evidence for NO_2_ after first violence exposure), supporting a theoretical model wherein individuals may become susceptible or “primed” through social pathways to some environmental triggers, including traffic-related pollutants. Despite limitations of our data sets, these results agree with evidence elsewhere that chronic stress may shape biologic response in early life (Wright et al. 2004a) and potentiate effects of air pollution through common physiologic systems.

Our findings contribute to the existing environmental justice literature by identifying potential changes in pollution susceptibility within communities affected by violence and other stressors. We also indicate ancillary effects of violence on children in urban communities, in addition to direct injury and post-traumatic stress. Sampson et al. (1997) identified associations among neighborhood deterioration, violence, and low collective efficacy (social control of neighborhoods through residents’ collective effort); we observed that a heightened susceptibility to pollution, associated with violence exposures or fear thereof, may lead to synergistic health effects of social and physical environmental conditions.

Although our findings are biologically plausible and highly suggestive, there are potential limitations in interpreting our models. A clear limitation is our small sample size for investigating multiplicative effects; despite the relatively high asthma prevalence (26%), a larger cohort would be required to consider interactions across numerous potential risk factors. The pollution model relies on NO_2_ data, commonly used as a marker of primary vehicular emissions, but not necessarily representative of all pollutants of interest. Road construction and airplane technology changes made it unclear *a priori* whether NO_2_ could be predicted by spatial indicators in this neighborhood, particularly using 1990 traffic data, though our model proved robust. We lacked complete residential history, likely creating some misclassification in a cohort with significant residential instability. However, we found no difference in asthma prevalence or exposures between movers and nonmovers; thus we expect misclassification to be nondifferential, biasing results toward the null and making our significant results more noteworthy.

The interpretation of our interaction model is complicated by the fact that behaviors may differ in violent neighborhoods, where parents often keep children indoors due to fear of violence. Therefore, children may be more exposed to indoor NO_2_, which is generally higher than outdoor NO_2_, and is influenced both by infiltration from outdoors and by smoking, gas stoves, and other sources which may be more prevalent in lower-income communities (Baxter et al. 2006; Spengler et al. 1983). Future studies should examine differential susceptibility to a wider range of pollution exposures, including indoor allergens and environmental tobacco smoke; although we accounted for maternal smoking, we did not examine the effect of other smokers in the home. Likewise, ETV may proxy for other social exposures responsible for the susceptibility effect we found; children witnessing violence may have greater family instability (Overstreet and Braun 2000) or may be directly victimized, potentially leading to injury-related susceptibilities.

We aimed to investigate whether a psychosocial stressor modifies pollution effects on asthma, and would prefer a long-term stress measure to corroborate the ETV scale. The CCDS elicits distress using a 6-month symptoms recall, inappropriate to our goal of capturing life-course stress. The correlation between CCDS and violence does, however, corroborate an association, supporting the plausibility of stress pathways to pollution susceptibility, and should be investigated further.

Reporting bias—generally underreporting by survivors, perpetrators, and witnesses—hampers quantitative violence research (Gordon and Riger 1991), particularly for domestic and intimate violence. Our questionnaire focused largely on within-community events, potentially more accurately reported. A prior analysis indicated that parents underestimate older children’s exposures, but are better corroborated for near-home events, potentially due to shared experience (Thomson et al. 2002). We were unable to examine direct victimization because very little was reported, owing either to low prevalence or underreporting, and thus we likely have some misclassification of true ETV. Most asthma cases were reported during longitudinal follow-up, limiting recall bias in diagnoses, but our retrospective violence report is subject to recall bias. To assess recall bias in violence reports, we asked the caregiver to report on asthma episodes triggered by violence; because very few parents associated violent events with asthma symptoms, recall bias in ETV by asthma status is unlikely.

Despite these limitations, our study provides evidence of a synergistic effect between social and physical factors in asthma etiology. The study also provides a model for retrospectively estimating traffic-related exposures, accounting for intracommunity heterogeneity and temporal trends using GIS. We were able to create temporally calibrated estimates using a spatially dense and temporally rich pollution data set of NO_2_ measurements collected throughout our epidemiologic study period. Though few studies will have access to such data, existing models provide insight about site characteristics influencing exposure variability, and spatially resolved satellite pollution data may prove useful in some settings (Liu et al. 2005).

In future studies, a wider and more frequently assessed suite of social stressors and perceived stress measures should be employed to examine stress trajectories over time and temporality in susceptibility to environmental triggers. The roles of social support and health beliefs in mediating the effects of perceived stress on health outcomes should be considered as buffers in the pathway from stressor to stress response, the latter of which may influence contaminant susceptibility and health (Chen et al. 2003). We observed no correlation between ETV and pollution levels, but suggest that longitudinal investigation of multiple exposures, and multiple pathways, are needed to further elucidate these effects. The spatial correlation across multiple exposures deserves greater investigation, and should be addressed in larger studies investigating spatial autocorrelations across, and potential synergies among, social and physical environmental factors.

## Figures and Tables

**Table 1:** Traffic indicators explored as predictors of monthly NO_2_ concentrations at sampling sites.

Indicator type	Unit
Cumulative density scores	
Unweighted traffic density within 50-, 100-, 200-, 300-, 500-m buffers	Vehicle-meters per day/m^2^
Kernel (inverse-distance)-weighted density: 50-, 100-, 200-, 300-, 500-m buffers	Vehicle-meters per day/m^2^
Density of urban roads (> 8,500 cars/day) within 200 m	Vehicle-meters per day/m^2^
Summary measures	
Total road length within 50, 100, 200, 300, 500 m	Meters
Total average daily traffic × length within 200 m	Vehicle-meters per day/m^2^
Distance-based measures	
Distance to nearest urban road (> 8,500 cars/day)	Meters
To nearest major road (> 13,000 cars/day)	Meters
To nearest highway (> 19,000 cars/day)	Meters
To nearest MHD-designated truck route	Meters
Characteristics of nearest major road	
Average daily traffic	Vehicles/day
Average daily traffic/distance to major road	(Vehicles/day)/m
Diesel fraction	Percent
Trucks per day	Vehicles/day
Trucks/distance to major road	(Vehicles/day)/m

**Table 2:** Characteristics of cohort participants.

Characteristic	Full cohort (*n* = 413)	Lifetime residents (*n* = 255)
Child		
Sex [female (%)]	50	53
Race (%)		
Hispanic	52	52
Caucasian	44	44
Asthma diagnosis (%)		
Overall	26	24
Low ETV–Low NO_2_	24	22
Low ETV–High NO_2_	26	21
High ETV–Low NO_2_	16	11
High ETV–High NO_2_	36	43
Age in 1997	6.8 ± 1.6	6.6 ± 1.6
Rasch ETV	0.60 ± 0.97	0.60 ± 0.96
NO_2_ year of diagnosis	27.5 ± 4.3	27.6 ± 4.3
Mother (%)		
Asthma diagnosis	7.7	7.0
Less than high school education	41	43
Smoker	25	22

Values are percent or mean ± SD.

**Table 3:** Land use regression modeling results for annual average NO_2_ at 13 sampling sites (*R*^2^ = 0.83).

	Overall estimate (*p*-value)
Year (categorical)	
1987	19.84
1988	23.06
1989	24.12
1990	21.97
1991	20.62
1992	19.02
1993	19.90
1994	21.29
1995	18.20
1996	17.98
1997	22.65
1998	24.14
1999	23.55
2000	21.45
2001	21.22
2002	17.35
2003	11.85
2004	10.64
Distance to major road (> 13,000 cars/day) (m)	−1.27 × 10^−3^ (< 0.0001)
Kernel traffic density within 500 m (VMT/day)	0.0775 (< 0.0001)
Population density (persons/km^2^)	1.086 × 10^−4^ (< 0.0001)

VMT, vehicle miles traveled. The year values are coefficients for yearly categorical variables.

**Table 4:** Multivariate model for asthma diagnosis [OR (95% CI)].

	Full cohort	Lifetime residents
Maternal asthma (ever diagnosed)	1.31 (0.58–2.96)	0.89 (0.29–2.74)
*In utero* tobacco smoke exposure	1.07 (0.44–2.58)	1.87 (0.53–6.57)
Maternal smoking since birth	1.10 (0.70–1.72)	0.85 (0.45–1.63)
Less than high school education	1.14 (0.71–1.81)	1.12 (0.60–2.07)
Child’s sex (female)	0.85 (0.54–1.34)	0.62 (0.34–1.14)
Child’s age (≥ 7 years)	1.44 (0.90–2.33)	1.06 (0.56–2.00)
High ETV	0.89 (0.56–1.43)	1.10 (0.59–2.04)
NO_2_ year of diagnosis: low ETV	0.99 (0.73–1.34)	0.85 (0.56–1.27)
NO_2_ year of diagnosis: high ETV	1.63 (1.14–2.33)	2.40 (1.48–3.88)

ORs for NO_2_ are associated with a 1-SD (4.3 ppb) increase.
